# The Effect of Medicaid Enrollment on Family Caregiving

**DOI:** 10.1093/geroni/igab046.1931

**Published:** 2021-12-17

**Authors:** Eric Jutkowitz, Peter Shewmaker, Derek Lake, Momotazur Rahman

**Affiliations:** 1 Brown University, Brown University, Rhode Island, United States; 2 Brown Universtiy, Brown University, Rhode Island, United States

## Abstract

Most people with long-term care needs rely on family caregivers. People with long-term care needs are also more likely to be eligible for Medicaid, which is the largest public payer of home and community based long-term care services. Whether enrolling in Medicaid compliments or substitutes for family caregiving is unknown. We linked Health and Retirement Study (HRS) respondents with their Medicaid enrollment data (2002-2012), to determine the effect of enrolling in Medicaid on family caregiving hours. We identified 130 people that participated in the HRS interview prior to enrolling in Medicaid in the same year (i.e., untreated) and 142 people that participated in the HRS interview after recently enrolling in Medicaid (i.e., treated). Untreated and treated respondents had similar demographic characteristics (age, sex, race). We estimated a series of inverse probability weighted linear regression adjusted models to determine the difference in monthly family caregiving hours between individuals that newly enrolled in Medicaid compared to people that had yet to enroll. We controlled for HRS respondents’ demographics, health care utilization, and nursing home utilization. HRS respondents interviewed after enrolling in Medicaid received 5.98 (95%CI: -27.60, 39.57) fewer monthly hours of family caregiving than respondents that had yet to enroll in Medicaid. HRS respondents interviewed after enrolling in Medicaid were not statistically more likely to receive any family caregiving (risk difference 0.05% 95%CI: -0.16, 0.06) than HRS respondents that had yet to enroll in Medicaid. Initial enrollment in Medicaid does not substitute for family caregiving.

